# Art in Dentistry

**Published:** 1888-07

**Authors:** J. A. Robinson

**Affiliations:** Jackson, Michigan


### Art in Dentistry.

BY J. A. ROBINSON, D.D.S., JACKSON, MICHIGAN.

[Read before the Michigan State Dental Association at Ann Arbor, Michigan, March 20, 1888.]

Art is a transcript of nature. The more perfectly it is copied, the higher will be the art. It has been asserted that the ideal is higher than the actual, but this can hardly be sustained, for we cannot go beyond or above nature even in our thoughts. Mr. Alcott who has just passed away said, that art was the reasoning of things without the matter. Art is as natural to the human mind as religion; and religion is found everwhere. Some one has said "that the spirit goes upward and never returns." So art is an aspiration that is always seeking higher ideal of perfection. Artists do not make art; it grows out of an ideal to make something more perfect and better. The mere mechanic performs his labor by geometrical rules, he does everything by the square, the compass and level; every thing he builds must be like something some one else has builded. Everything in nature is built on strictly geometrical rules, but all things in nature differ from each other. It is in this grand variety that the greatest harmony is gained.

There is a great significance to the term "tooth carpenters" that we so often hear used to express the person who has a sign over his office saying *Dentist*, when he is merely a *mechanic*; and prosthetic dentistry expresses more fully our calling in supplying artificial dentures. The dentist has two great laws to fulfill. One is *form or condition*, and the other is *color*. In everything he does he is obliged to work under circumscribed conditions. First, the face or general contour of expression must be preserved or restored; and next, each individual tooth must possess character and life within itself. One would hardly think that all things in nature so closely correspond; the median and distil points are as largely in correspondence with the centre as the leaves we find on all forest trees. The flowers (which are only modified leaves) all have this beautiful one-sided harmony of nature that she has bestowed upon each individual tooth, and of the superior and inferior maxillary as a whole. This rule is to be observed not only in supplying artificial dentures, but in filling teeth with whatever substance is used to complete the work. It is within the region of probability that the dental art will arrive to such a degree of excellence within the next few years, that the higher art will be realized of preventing decay and restoring the natural organs to such a degree of usefulness that we shall hear no more about tedious and painful operations; and that extracting teeth, as Mr. Carlisle would say, will become an unpleasant reminiscence.

When we become a profession of artists we shall hear no more about the cheap dentist, and running down prices, for the real artist is so imbued with the love of his calling that he is above the little bickerings with his neighbors; but in his endeavor to reach his highest ideal, his pocket and his ledger will be the least of his concern about his work. This is one of the tests of perfect art and of the real artist to-day. Another test is to be found in pleasurable emotion. The satisfaction we experience in accomplishing and overcoming difficulties and imitating nature; and carrying art to the anticipated perfection will correspond to the satisfaction we experience in listening to the finest music or oratory, or of viewing the most perfect painting or statuary. When that time dawns upon us as the light we have already foreshadowed some of you may live to see, we shall be fit to be called *dentists*, and shall be *artists indeed.*

Art is made up of seeing, thinking, and doing. A lack of either faculty destroys our usefulness in art, and we find imperfection of finish in the work we undertake to perform.

If it be true as has often been asserted that dentistry has made greater progress and advancement in the arts and sciences than either of the older professions, it may be accounted for by the fact that we have never been surrounded and permeated by those conventionalities that are incident to time-honored and moss-grown institutions that retard their progress. I allude particularly to the public press as a means of information, education and culture of the masses in this nineteenth century. The suppression of truth and information, and the love of money getting are the instruments that strangle our progress in true art and culture, and the advancement of our free institutions. The perils alluded to will destroy our usefulness and progress. No institution or profession can withstand such an ordeal as the suppression of public information from within, and the trusts and combinations from without.

DISCUSSION.

*Dr. J. Taft:*—The paper that has just been read is of interest to us all. As you all know, there is a difference of opinion in regard to spreading the truth among people. Some favor one method, some another. Each claims his to be the best. The broader the culture of the people, the less occasion is there for any special presentation of truth in regard to the profession. That which the well prepared dentist is capable of doing will makes a far better and far more lasting impression than what he may say of himself through the press. He will impress the public by his character. The good words of a newspaper last but a day. They are soon forgotten. A man's own work is that which makes the most lasting impression. It may have been important some time ago to let the public hear from us and about ourselves in the papers—to especially enlighten them on dentistry; but it seems to me the case is now different. Is it necessary for a well accomplished young man to resort to special methods?

*Dr. A. T. Metcalf:*—To bring the matter down to facts, let us see who are the men that advertise. Who is the professional, or rather so-called professional doctor that comes to your city and informs you of it with flaming posters? Is he not often one who has been a failure at home? I have never been so poor a man or dentist that I have had to advertise my wares. The people will find us without any advertising. I am astonished that any one who has been a member of the Michigan Dental Association, who has been with us so long, should have just discovered that he must advertise! I can to a certain extent overlook it in one of the members since he has a patent from which by advertising he can obtain a few cents. Yet it is too much of the style of a quack to do this. It is the quack who resorts to such methods.

*Dr. R. Douglass:*—A man who does good work advertises himself. Several years ago the Michigan Dental Association circulated a pamphlet in regard to dentistry. We should do so again. But for a man to extol himself above his fellow dentists is wrong. To say that there are but two or three others in the State who can perform a certain operation, does not lift the profession. A pamphlet like the one formerly printed should be distributed so as to let people know what can be done in the way of saving their teeth. It is a hard matter to persuade people that dentists can do any thing but pull out their teeth.

*Dr. J. A. Watling:*—I remember the pamphlet referred to. I am sure it did a great deal of good. But it was not an advertising dodge to get business. It had no name on it; advertised no one. But the advertising dentist claims he is different from others. He can do this or he can do that in a more easy and better way. If a doctor come among such people as do not know what dentists do, he had better move away. Now, advertising may be a very profitable investment as far as money is concerned, but stick to your office, do good work, and it will not be long before people will recognize your worth.

*Dr. J. Taft:*—We wish to consider the best means of making the public acquainted with what we can do for them. There are certain things we should remain silent about. Do not talk of the different operations and such things as few can well understand. But tell of the care to be taken of the teeth. Tell what can be done, but do not mount a pedestal and say, "I alone can do this or that!" In this we find the great objection to advertising. *Ego* is too apparent. It is all *ego*. Then nineteen-twentieths of the advertisements contain more or less of untruth. They are ordinarily for the money consideration. They are too mercenary. And more, though a man tell the truth and only the truth, he is after all in bad company. There are quack advertisements on every side of his. See the style of the advertisements. Keep out of such company. There are many and enough things to tell your patients: how to take care of themselves, etc. Suppose you do let the public judge you. Advertising tells against you. Discriminating people will compare you with other advertisers. Build up your business with good work so that you may years afterwards stand with a world-wide reputation.

*Dr. J. A. Robinson:*—The great men of all times have been those of the greatest personality. I am not an advertiser myself, but I am in favor of the truth. The man who tells the reporter of his great operation advertises. We should be consistent. I believe in teaching by the sight. You all agree with me there. Thus we put diagrams in the papers. I never did it, but I always advocate the support of the truth.

